# Tomato growth stage modulates bacterial communities across different soil aggregate sizes and disease levels

**DOI:** 10.1038/s43705-023-00312-x

**Published:** 2023-09-26

**Authors:** Menghui Dong, Eiko E. Kuramae, Mengli Zhao, Rong Li, Qirong Shen, George A. Kowalchuk

**Affiliations:** 1 Key Lab of Organic-Based Fertilizers of China, Jiangsu Provincial Key Lab for Solid Organic Waste Utilization, Jiangsu Collaborative Innovation Center of Solid Organic Wastes, Educational Ministry Engineering Center of Resource-Saving Fertilizers, Nanjing Agricultural University, Nanjing, 210095 Jiangsu China; 2 Ecology and Biodiversity Group, Department of Biology, Institute of Environmental Biology, Utrecht University, 3584 CH Utrecht, The Netherlands; 3 Department of Microbial Ecology, Netherlands Institute of Ecology, 6708 PB Wageningen, The Netherlands; 4 State Key Laboratory of Subtropical Silviculture, Zhejiang A&F University, Hangzhou, 311300 China

**Keywords:** Microbial ecology, Microbiome

## Abstract

Soil aggregates contain distinct physio-chemical properties across different size classes. These differences in micro-habitats support varied microbial communities and modulate the effect of plant on microbiome, which affect soil functions such as disease suppression. However, little is known about how the residents of different soil aggregate size classes are impacted by plants throughout their growth stages. Here, we examined how tomato plants impact soil aggregation and bacterial communities within different soil aggregate size classes. Moreover, we investigated whether aggregate size impacts the distribution of soil pathogen and their potential inhibitors. We collected samples from different tomato growth stages: before-planting, seedling, flowering, and fruiting stage. We measured bacterial density, community composition, and pathogen abundance using qPCR and 16 S rRNA gene sequencing. We found the development of tomato growth stages negatively impacted root-adhering soil aggregation, with a gradual decrease of large macro-aggregates (1–2 mm) and an increase of micro-aggregates (<0.25 mm). Additionally, changes in bacterial density and community composition varied across soil aggregate size classes. Furthermore, the pathogen exhibited a preference to micro-aggregates, while macro-aggregates hold a higher abundance of potential pathogen-inhibiting taxa and predicted antibiotic-associated genes. Our results indicate that the impacts of tomatoes on soil differ for different soil aggregate size classes throughout different plant growth stages, and plant pathogens and their potential inhibitors have different habitats within soil aggregate size classes. These findings highlight the importance of fine-scale heterogeneity of soil aggregate size classes in research on microbial ecology and agricultural sustainability, further research focuses on soil aggregates level could help identify candidate tax involved in suppressing pathogens in the virtual micro-habitats.

## Introduction

Plant-microbe interactions greatly impact agricultural production and protection [1–3]. Plant roots significantly impact the physical and chemical properties of the microhabitat in soil in which microorganisms reside [4], leading to changes in the microbial community composition in the rhizosphere, and even bulk soil [5]. These changes in microbial communities, in turn, have important feedback on plant performance [4, 6, 7]. A better understanding of how plant roots impact soil structure and microbial communities could contribute to improving sustainable agricultural production.

Soil structure regulates many processes in soils, including water flow, gas exchange, nutrient cycling, root penetration, and microbial activities [8]. Soil aggregation is a key element of soil structure where micro-aggregates (<250 μm) are formed by primary particles (<53 μm) and humus, and macro-aggregates (>250 μm) are further formed by organic polymers, fungal hyphae, and plant root [9, 10]. In the last decades, most research has examined soil aggregation from the perspective of soil management [11–14] and its impact on ecosystem processes such as soil carbon storage [15, 16]. Moreover, it is crucial to recognize that soil aggregation plays a significant role in shaping the composition and functioning of the soil microbiome as the heterogeneous micro-environments within different size classes of soil aggregates support varied microbes and their activities [8, 17, 18].

Plant roots have a direct impact on root-adhering soil aggregates, making them a crucial focus when studying plant-soil feedback. Previous studies have contributed to our understanding of how root-associated microbiota impact the aggregation of root-adhering soil [19–22]. However, very few studies have addressed how plant impacts, such as priming effects, differentially impact microbial community activities across different soil aggregates [23]. Plant roots can have differential effects on microbes residing in different size classes of soil aggregates due to the heterogeneity in physio-chemical properties. For instance, the soil organic matter (SOM) within soil aggregates that support microbes differ across size classes; macro-aggregates contain fresh SOM, whereas micro-aggregates enclose older organic matter [24]. These divergences in organic matter among soil aggregates culminate in dissimilar responses of microbiomes within distinct size classes to root exudates, attributable to variations in chemical composition. Nonetheless, there is limited knowledge about how microbial residents in different particle size classes are impacted by plants during different growth stages, particularly regarding their role in pathogen suppression. The soil-borne pathogens and their inhibitors might exhibit preferences for specific habitats created by the heterogeneity of different soil aggregate size classes. For example, Fe is closely linked to plant pathogens [25, 26], and the distribution of available Fe was reported to vary among soil aggregate size classes [27], which can lead to a preference of residence for pathogens. Evidence has shown that the improvement of macro-aggregate formation may induce soil conduciveness of banana *Fusarium* wilt by increasing the available Fe content [28], which is necessary for the germination of the spores of *Fusarium*, but the distribution of the pathogen among soil aggregates was not assayed. Current studies often treat soil as a homogeneous habitat, disregarding the fine-scale heterogeneity within soil aggregates where microbial interactions occur. This approach leads to a loss of information about the spatial distribution of individual microbes and in-situ microbial interactions during sample processing [18]. By recognizing the fine-scale heterogeneity of soil aggregates, we can identify potential taxa involved in suppressing soil pathogens and develop management strategies to stimulate these target populations.

**Fig. 1:**
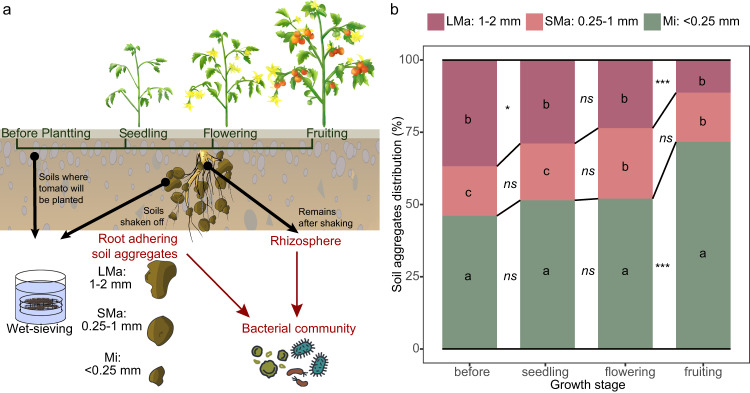
Feild experiment description and soil aggregates distribution. **a** Experimental design. **b** Soil aggregates distribution in different growth stages. The asterisk indicates a statistically significant difference between two growth stages in each individual soil aggregate size class: ns no significant difference; *p < 0.05; **p < 0.01; ***p < 0.001. The lowercase letters indicate the significant difference between size classes in individual stages. The significances were determined by Compute Tukey Honest Significant Differences test (TukeyHSD) based on Two-way ANOVA. Stacking bars represent the average of six replicates.

In this study, we examined the impact of plants on soil structure and bacterial community composition, succession, and potential function across different soil aggregate size classes to demonstrate the effect of fine-scale heterogeneity of soil aggregates on modulating the impact of the plant on soil bacterial communities. We also examined whether the distribution of specific taxa across different soil particle size classes corresponded to the density of a soil-borne plant pathogen. We used long-term tomato fields as a model to explore how bacterial residents of different particle size classes are impacted by plants throughout their growth stages (Fig. 1a). The field has been continuously cultivated with tomato *Solanum lycopersicum* L. for seven cropping cycles, leading a build-up of bacterial wilt disease caused by *Ralstonia solanacearum*. This allowed us to evaluate the potential role of the microbiome within soil aggregates in disease suppression. We collected root-adhering soil aggregates at different growth stages (before-planting, seedling, flowering, and fruiting) and separated them into size classes (large macro-aggregates, LMa, 1–2 mm; small macro-aggregates, SMa, 0.25–1 mm; Micro-aggregates, Mi, <0.25 mm). In addition, we took root-adhering soil aggregate samples and rhizosphere samples. The root-adhering soil aggregate samples were obtained by wet-sieving the soils that were loosely bound to the tomato roots. This procedure aimed to illustrate how tomato roots influence the composition, progression, and potential roles of bacterial communities within distinct soil aggregates across tomato growth stages. On the other hand, the rhizosphere samples encompass tightly remaining soils after root shaking and are collected by root washing. This step was undertaken to examine the dynamics of the *Ralstonia* pathogen and its potential inhibitory taxa within the rhizosphere, while also tracing their distribution across different soil aggregate size classes. All samples had DNA extracted and the DNA was subjected to qPCR and Illumina MiSeq sequencing based on the 16 S rRNA gene to quantify the bacterial density and community composition. We hypothesized that (1) different size classes of root-adhering soil aggregate would differ in terms of total bacterial density, community composition, and specific ASVs (amplicon sequence variants), and (2) the potential functioning of soil aggregates in pathogen inhibition would vary among size classes, as related to their differences in bacterial community structure and potential function.

## Materials and methods

### Field site description

The experimental tomato fields were located at the Nanjing Institute of Vegetable and Flower Science, Nanjing, China (32°02’N, 118°50’E). This region has a tropical monsoon climate with an average annual temperature and precipitation of 15.4 °C and 1106 mm, respectively. Fields have been continuously cropped with tomato (*Solanum Lycopersicum* L.) since March 2014 with two cropping cycles per year, one in the Spring and one in the Autumn. The field was protected by a plastic shed, which included three independent field plots. These field plots had the same management: regularly irrigated during tomato cultivation, tillage, and fertilization, and the roots and shoots of tomatoes were removed after the harvest. Both organic and chemical fertilizers were applied to the fields with a total of 7500 kg/ha (dry weight) organic fertilizer fermented by chicken manure, and chemical fertilizers including urea, calcium superphosphate, and potassium sulfate applied supplementally to adjust the inputs in a total amount of N (120 kg/ha), P (180 kg/ha) and K (120 kg/ha) in the sample collection season.

With the long-term monoculture of tomatoes, these fields suffered seriously from tomato bacterial wilt caused by a soil-borne pathogen *Ralstonia solanacearum* [29, 30]. In the 7th cropping cycle from which we collected the samples, the disease incidence of these field plots ranged from approximately 21.4% to 68.8% [31] based upon visible symptoms of tomato bacterial wilt (e.g., initial wilting of upper leaves followed by complete wilting of all leaves) [32].

### Soil sampling

Soils were sampled in spring 2017 during the 7th cropping cycle. Soil samples were collected from 4 growth stages of the tomato, i.e., before-planting, seedling, flowering, and fruiting stage at weeks 0, 2, 5, and 10, respectively (Fig. 1a). To avoid the impact of irrigation time, soil samples were collected 3 days after irrigation at each time point. Three independent field plots were split into six subplots in the middle (two subplots for each field plot) as subplots for sampling. Before tomato planting, soil cubes (10× 10× 10 cm) were sampled randomly from 3 distinct locations where tomatoes will be planned within each subplot and pooled as one biological sample. After tomato planting, 3 healthy plants of tomato were randomly selected from each subplot for each growth stage (seedling, flowering, and fruiting). The roots of these plants were dug up together with surrounding soils (in 1000 cm^3^ cubes), and these soil cubes were transported to the lab and immediately stored at 4 °C. The 3 plants or soil cubes collected from each subplot were pooled as one sample. In total, 6 replicate samples were collected for each stage. Soil moisture was determined by the oven-drying method. For samples from the before-planting stage, soil cubes were gently broken up by hand along natural planes of weakness and passed through an 8 mm sieve, after which soils were directly subjected to a wet sieve for soil aggregate separation. For samples from the other 3 growth stages with roots, soil cubes were gently broken by hand along natural planes of weakness, and soils that didn’t adhere to the roots were removed. Then, roots were shaken fiercely by hand, and soil that had shaken off from the roots was collected and subjected to aggregate separation to create the root-adhering soil aggregate samples. The rhizosphere samples were provided by Zhao [33]. Briefly, soils that remained on the root after the shake were collected by root wash [30, 34].

### Soil aggregate separation

Soil aggregates were separated by a modified wet-sieving method [35, 36]. In brief, fresh soils were air-dried in the lab until the soil moisture was around 20% (60% of field capacity) to avoid the impact of soil moisture in the determination of soil aggregate size distribution. Then, 25 g of soil was wet sieved by a column of sieves including 2 mm, 1 mm, and 0.25 mm. Soils were placed on the top of the sieve column and immersed in sterilized water for 5 min. Then, the sieves were gently shaken 50 times by hand over the course of 2 min with an amplitude of 4 cm. The soil remaining on each of the sieves was air-dried for about 3 min under a sterile wind to make it of suitable moisture for collection. The size classes passing through the 0.25 mm sieve were collected by centrifugation (6000 g/min, 10 min). A part of the collected size classes was oven-dried at 60 °C for 24 h to determine soil moisture, and the remainder was stored at −80 °C for DNA extraction.

Since no soil remained on the top of the 2 mm sieve, root-adhering soil aggregate samples were named as follows: Large macro-aggregates (LMa), 1–2 mm; Small macro-aggregates (SMa), 0.25–1 mm; Micro-aggregates (Mi), <0.25 mm.

### 16 S rRNA gene amplicon sequencing

Total soil DNA was extracted by PowerSoil DNA Isolation Kit (Mobio Laboratories Inc., Carlsbad, USA) using 0.5 g dry weight soil of each soil sample, following the manufacturer’s instructions, and stored at −80 °C.

The V4 hypervariable region of the 16 S rRNA gene (~283 bp) was amplified with primers 520 F (5′-AYTGGGYDTAAAGNG-3′) and 802 R (5′-TACNVGGGTATCTAATCC-3′) [33]. PCR amplicons from total soil DNA (as described in Supplementary Information) were subjected to DNA sequencing using an Illumina Miseq PE250 platform (Illumina Inc., CA, US) at Personal Biotechnology Co., Ltd. (Shanghai, China) to determine the bacterial community compositions. Raw sequence data is available in the National Center for Biotechnology Information (NCBI: https://www.ncbi.nlm.nih.gov/) with the BioProject accession number PRJNA911225. The raw rhizosphere amplicon sequencing data was obtained from Zhao [31], which was processed using the same protocol for DNA extraction and MiSeq sequencing, as described in the Supplementary Information.

### Bioinformatic analysis

Raw paired-end reads of soil aggregate and rhizosphere samples were processed by QIIME2-2021.8 [37] according to the pipeline. DADA2 [38] was performed by the “dada2 denoise-paired” function to determine the amplicon sequence variants (ASVs). Forward and reverse reads were trimmed at the 5′ end until 15 and 18 bp, respectively, to remove the primers and truncated at 3′ end until 180 and 160 bp, respectively, to remove the low-quality base pairs. Then, contingency-based filtering was performed to filter the ASVs that show up in no more than 5 samples using the “feature-table filter-features” function. At last, a total of 3,724,170 high-quality and non-chimeric sequences were obtained from a total of 114 samples, with a median of 30,345 sequences per sample (ranged from 18,477 to 52,667). Rarefaction curve (Supplementary Information Fig S1) was performed to evolute the intensity of sampling, using “rarecurve” function of “vegan” package in R 4.1.1. The rarefaction curves reached their asymptotes or started to plateau for all samples, suggesting that saturation in sequencing was achieved. Taxonomy was classified using a pre-trained Naïve Bayes classifier by “feature-classifier” function against the Silva Ref NR99 [release 132] database [39].

To predict the potential functions based on 16 S rRNA gene sequences, we applied PICRUSt2 (version 2.4.1) [40] based on the ASV abundance and sequence table using the “picrust2_pipeline.py” function with default parameters. The predicted KEGG Orthology (KO) dataset was used to compare the difference between macro and micro-aggregates in potential functions. The KOs were annotated according to the KEGG ORTHOLOGY Database (https://www.genome.jp/kegg/ko.html) [41].

### Real-time qPCR

To determine the density of bacteria within each soil aggregate size class across all growth stages, the real-time qPCR test was performed based on part of the V3 region of the 16 S rRNA gene (primer 338 F 5′-ACTCCTACGGGAGGCAGCAG-3′ and primer 518 R 5′-ATTACCGCGGCTGCTGG-3′) [42]. We also quantified the population density of the tomato bacterial wilt pathogen, *R. solanacearum*, within each size class in the fruiting stage while the disease breaks out, based on *filC* gene (primer F 5′-GAACGCCAACGGTGCGAACT-3′ and primer R 5′-GGCGGCCTTCAGGGAGGTC-3′) [43]. Protocols of the real-time qPCR were shown in Supplementary Information.

### Statistical analyses

All statistical analyses were performed in R version 4.1.1 (R Core Team 2021). For alpha-diversity analyses, the total numbers of sequences in each sample were rarefied to an equal sum of 18,477 (minimum sum over all samples) using a “Rarefy” function in “GUniFrac” package. Beta-diversity analyses were based on a non-rarefied dataset. The dataset was Hellinger-transformed, Bray–Curtis dissimilarity was calculated based on the transformed dataset. Principal coordinate analysis (PCoA) was used to visualize the dissimilarity matrices. Significant variation between bacterial communities was tested by analysis of similarities (ANOSIM) with 999 permutations using “anosim” function of package “vegan” based on Bray–Curtis dissimilarities. “DESeq2” and “edgeR” package were employed to identify the significantly different bacterial taxa between successive growth stages in each soil aggregate size class. To ensure the robustness of results, we focused on the intersection of ASVs that possessed adjusted *P*-values lower than 0.05, as determined by both DESeq2 and edgeR. Then Venn network diagrams were drawn in Cytoscape (Version 3.9.1) to show their associations. The significant difference between values such as bacterial density, alpha-diversity, dissimilarities, etc. was calculated by Tukey Honest Significant Differences (Tukey HSD). Correlation tests were performed by Spearman’s correlation test. Comparison between the relative abundance of ASVs or density of *R. solanacearum* was performed by Wilcoxon Rank Sum and Signed Rank Tests. For predicted functions, the values of KOs were rounded into integers, and then, genes enriched in macro or micro-aggregates were determined by DESeq2. KOs that had *p*-values less than 0.01 and absolute values of log2FoldChange more than 1.5 were defined as enriched KOs.

## Results

### Soil aggregate distribution changed with tomato growth stages

The distribution of soil aggregates showed dynamic changes across the different plant growth stages (Fig. 1b). Micro-aggregates (Mi) showed the highest abundance (*p* < 0.05) in all stages compared to other macro-aggregate size classes (i.e., LMa and SMa). The abundance of Mi increased with the development stage, especially in the fruiting stage, with a high level of significance (*p* < 0.001) detected. In contrast, the proportion of large macro-aggregates (LMa) was highest before planting. This size class decreased with plant development, with a non-significant decrease (*p* > 0.1) from the seedling to the flowering stage, and a highly significant decrease (*p* < 0.001) from the flowering to the fruiting stage. The proportion of the SMa size class showed no significant changes between growth stages. In short, the planting and growth of tomatoes negatively impacted root-adhering soil aggregation.

### Bacterial density changes showed markedly different dynamics in micro-aggregates as compared to macro-aggregates

**Fig. 2:**
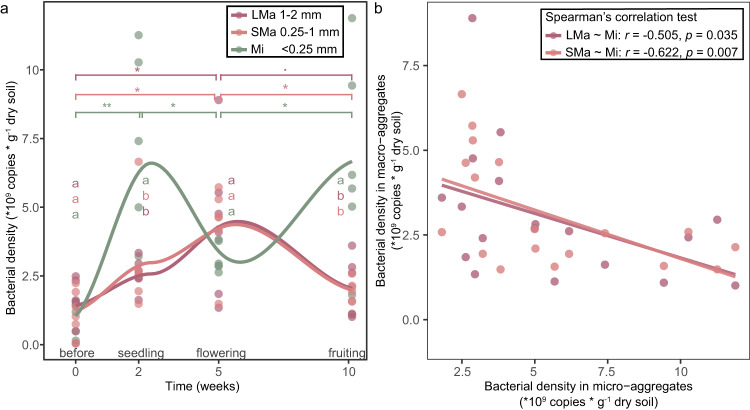
Dynamic of bacterial density in different soil aggregate size classes. **a** Bacterial density in soil aggregates based on the real-time qPCR test. **b** Negative correlation in bacterial density between micro-aggregates and macro-aggregates. LMa large macro-aggregate, 1–2 mm; SMa small macro-aggregate, 0.25–1 mm; Mi micro-aggregate, <0.25 mm. The asterisk indicates a statistically significant difference between two growth stages in each individual soil aggregate size class: ‘*p* < 0.1; *p < 0.05; **p < 0.01. The lowercase letters indicate the significant difference between size classes in each individual stage. The significances were determined by Compute Tukey Honest Significant Differences test (TukeyHSD) (*p* < 0.05) based on Two-way ANOVA. The correlation was determined by Spearman’s rank correlation test based on density data excluding the before stage.

To investigate the impact of soil aggregation on bacterial density, real-time qPCR was performed to track the bacterial density within soil aggregates for each growth stage (Fig. 2a). The bacterial densities were similar (*p* > 0.1) between soil aggregates before tomato transplanting. The bacterial density in micro-aggregates (Mi) significantly (*p* < 0.05) increased in the seedling stage, causing a significantly higher density in Mi as compared to LMa and SMa. In the fruiting stage, the bacterial density in macro-aggregates decreased (i.e., *p* < 0.1 in LMa, *p* < 0.05 in SMa), while the density in micro-aggregates significantly increased (*p* < 0.05), causing a significant (*p* < 0.05) higher density in Mi than LMa and SMa. These results indicated that bacterial density showed a similar dynamic change in macro-aggregates (i.e., LMa and SMa), but an opposite pattern in micro-aggregates compared to macro-aggregates. Spearman’s rank correlation test also showed that the bacterial density in stages after tomato transplanting (i.e., seedling, flowering, fruiting) showed a significantly negative correlation (i.e., LMa~Mi, *r* = −0.505, *p* < 0.05; SMa~Mi, *r* = −0.622, *p* < 0.01) between Mi and LMa or SMa (Fig. 2b), again indicating opposite patterns of bacterial density in macro-versus and micro-aggregates for root-adhering soil.

### Impact of soil aggregates on beta-diversity of bacterial community succession

To show the variation among aggregate size classes and growth stages, principal coordinate analysis (PCoA) was performed based on Bray–Curtis. When all samples were included in the analysis (i.e., rhizosphere, whole soil, and root-adhering soil aggregates) (Supplementary Information Fig S2), both the size class and the growth stage significantly (*p* < 0.001) impacted the bacterial composition according to ANOSIM. The soil aggregate size explained a higher degree of variation (*r* = 0.36) compared to the growth stage (*r* = 0.245). The PCoA also showed a clear distinction between rhizosphere and root-adhering soil.

**Fig. 3:**
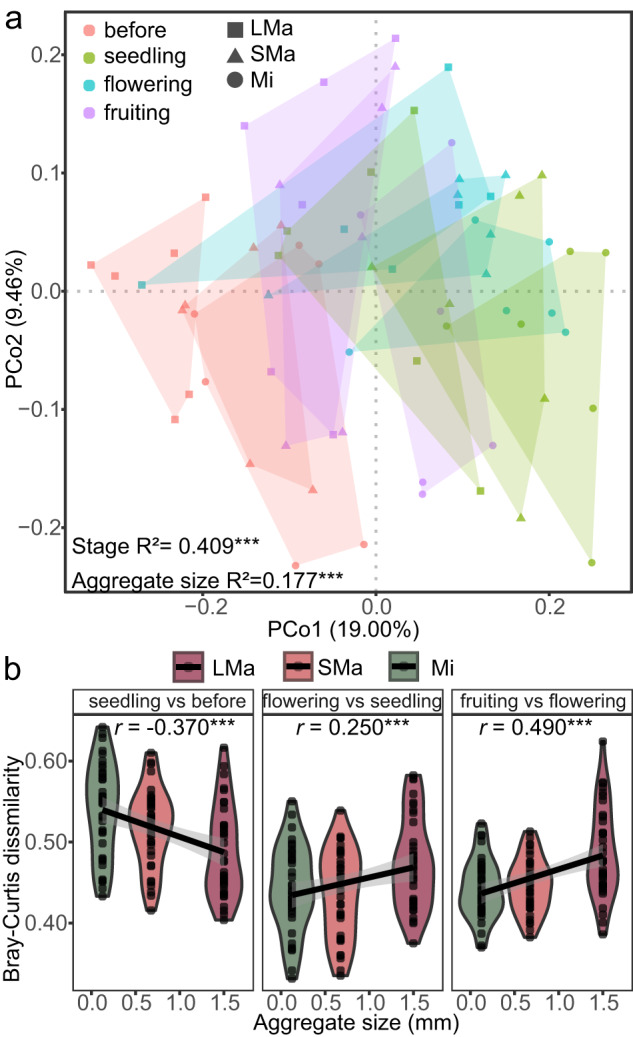
Bacterial community composition and turnover. **a** Principal coordinates analysis (PCoA) and Analysis of similarity (ANOSIM) based on Bray–Curtis dissimilarity. LMa large macro-aggregate, 1–2 mm; SMa small macro-aggregate, 0.25–1 mm; Mi micro-aggregate, <0.25 mm. The colors mean different growth stages, and shapes indicate the size classes of soil aggregates. *** indicates significant difference (*P* < 0.001) according to ANOSIM. **b** Bray–Curtis dissimilarities of successive growth stages in individual soil aggregate size classes and its correlation with soil aggregate sizes. The correlations were determined by Spearman’s correlation test, *** means the significance level of correlation (*P* < 0.001).

To further evaluate the impact of root-adhering soil aggregates on bacterial community succession, we performed PCoA and ANOSIM using soil aggregate samples based on Bray–Curtis dissimilarity (Fig. 3a). The PCoA revealed a clear separation between the soil aggregate size classes and growth stages, while ANOSIM also indicated that the growth stage and soil aggregate size significantly impacted the bacterial community composition. In this case, growth stage explained more variation (*r* = 0.409) than soil aggregate size class (*r* = 0.177). Soil aggregate size showed various effects on bacterial community succession across the different tomato growth stages. Bray–Curtis dissimilarities were extracted between each of the two continuous growth stages in individual soil aggregate sizes (Fig. 3b) to examine bacterial community succession in each soil aggregate size class. The soil aggregate sizes showed a negative correlation (*r* = −0.37, *P* < 0.001) with Bray–Curtis dissimilarities in the seedling stage *versus* the before-planting stage, indicating a greater change in smaller size classes in the seedling stage. In contrast, soil aggregate size showed positive correlations with Bray–Curtis dissimilarities in later growth stages (flowering *vs* seedling: *r* = 0.25, *P* < 0.001; fruiting *vs* flowering: *r* = 0.49, *P* < 0.001), suggesting that greater changes occurred in larger size classes in the flowering and fruiting stages.

### Taxa affected by successive growth stages in each soil aggregate size class

**Fig. 4:**
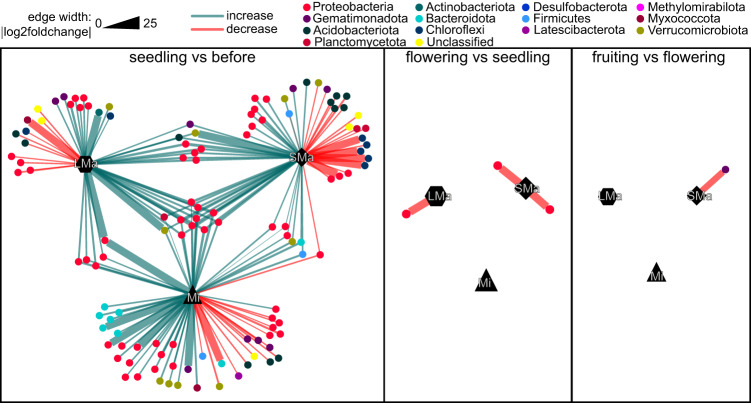
Venn network showing the distribution of significantly changed ASVs between two successive growth stages in individual soil aggregate size classes. LMa large macro-aggregate, 1–2 mm;  SMa small macro-aggregate, 0.25–1 mm;  Mi micro-aggregate, <0.25 mm. The circles in different colors represent ASVs classified into different phyla. Edges between soil aggregate size classes and ASVs indicate these ASVs significantly changed in that soil aggregate size class according to both DESqe2 and EdgeR. Edges in green mean a significant (*p*_adjust_ < 0.05) increase according to DESeq2, while edges in red mean a significant decrease (*p*_adjust_ < 0.05). The edge width represents the absolute value of log2 fold change calculated by Deseq2.

To investigate the ASVs that significantly changed between two successive growth stages in each soil aggregate size class, DESeq2 and edge R were performed, and Venn network diagrams were drawn to show their associations (Fig. 4). The greatest change occurred between the seedling and before-planting stages, with a total of 121 ASVs significantly changed (i.e., 74 increased, 47 decreased), while only a few ASVs significantly changed in the flowering (i.e., 0 increased, 3 decreased) and fruiting (i.e., 0 increased, 1 decreased) stages. Interestingly, in the seedling stage, most of the affected ASVs were unique to each soil aggregate class (i.e., 90 unique ASVs; 31 common ASVs), indicating a disparate change of ASVs in different soil aggregates. Almost all (i.e., 30 out of 31) of the common ASVs increased between the before-planting and seedling stage and a majority (i.e., 23 out of 31) of these ASVs belong to the Proteobacteria, indicating an overall effect of root compounds on enriching similar taxonomic groups in all soil aggregate size classes. In the flowering and fruiting stages, all significantly changed ASVs were unique to each soil aggregate size class, indicating that roots had a distinct effect on ASVs in soil aggregates along the growth stages.

### The potential function of pathogen inhibition across soil aggregate size classes

**Fig. 5:**
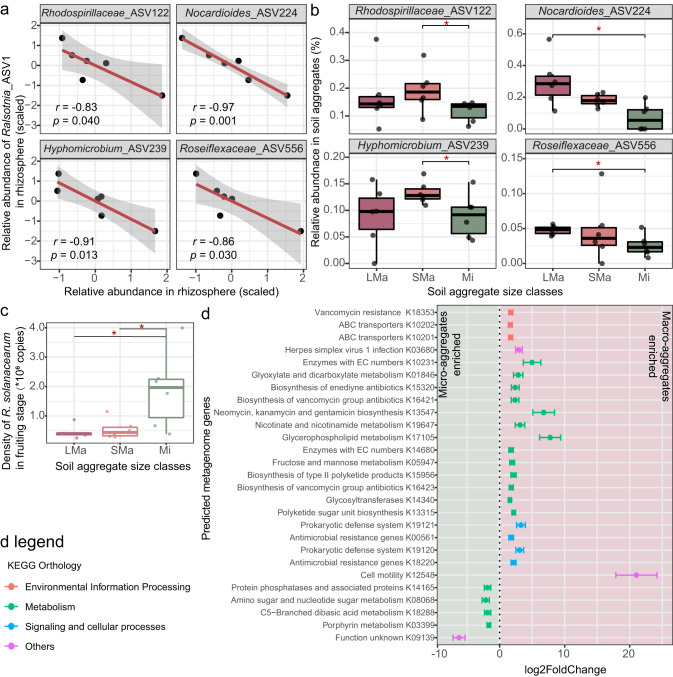
Potential microbial function for pathogen suppression across soil aggregate size classes. **a** ASVs that negatively correlated with *Ralsonia*_ASV1 in the rhizosphere. Correlations were determined according to Spearman’s correlation test. **b** The distribution of these four negatively correlated ASVs among soil aggregate size classes. Asterisks indicate a significant difference (*p* < 0.05) according to Wilcoxon Rank Sum and Signed Rank Tests. LMa large macro-aggregate, 1–2 mm; SMa small macro-aggregate, 0.25–1 mm; Mi micro-aggregate, <0.25 mm. **c** The Density of *R. solanacearum* among soil aggregate size classes in the fruiting stage according to qPCR. Asterisks indicate a significant difference (*p* < 0.05) according to Wilcoxon Rank Sum and Signed Rank Tests. **d** Predicted metagenome genes that enriched in micro or macro-aggregates in the fruiting stage. Deseq2 were used to determine the enrichment, KOs with *p* < 0.01 and |log2FoldChange | > 1.5 were defined as enriched KOs.

After long-term continuous cropping of tomatoes, the experimental fields suffered seriously from tomato bacterial wilt caused by *R. solanacearum*. In the 7th cropping cycle, the one from which we collected our samples, the disease incidence of the three plots reached approximately 21.4%, 50%, and 68.8%, respectively. Bacterial wilt symptoms typically broke out at the fruiting stage. It is also at this time that we saw an explosive increase of ASV1 (Supplementary Information Fig S3). This ASV had a high mean relative abundance of 64.02% in the fruiting rhizosphere and was classified as being affiliated with the *Ralstonia* genus. We, therefore, assumed that this ASV corresponds to the population causing the bacterial wilt in these fields. To examine if ASVs could be identified that might be involved in pathogen suppression, we searched for ASVs that were negatively correlated with the pathogen in the rhizosphere. To this end, we performed a Spearman’s rank correlation test to determine the ASVs that had a significantly negative correlation (*p* < 0.05) with the relative abundance of the *Ralstonia* ASV1 in the fruiting rhizosphere. A total of 29 such ASVs were found. Then we investigated the distribution of these potential pathogen-suppressive ASVs across soil aggregate classes, and 21 of these ASVs were found in soil aggregate samples (relative abundance of these ASVs shown in Supplementary Information Fig S4). Of these ASVs, 4 were found to vary significantly in relative density between soil aggregate size classes. Figure 5a shows the correlations of these 4 ASVs with the *Ralstonia*_ASV1. All these 4 ASVs had a significantly (*p* < 0.05) higher relative abundance in one of the macro-aggregate size classes as compared to the micro-aggregate size class (Fig. 5b). Two of them (i.e., ASV224 and ASV556) had a significantly (*p* < 0.05) higher abundance in LMa than Mi size class, and the other two (i.e., ASV122 and ASV239) were significantly (*p* < 0.05) higher in SMa than Mi. The above results suggest that macro-aggregates have more potential pathogen inhibitors compared to micro-aggregates in the growth stage where the disease becomes manifest.

To further investigate the potential function of the bacterial community in soil aggregates on pathogen inhibition, the density of the pathogen *R. solanacearum* in the fruiting stage was examined by qPCR test (Fig. 5c). Interestingly, the density of *R. solanacearum* was significantly higher in the Mi size class than LMa and SMa size classes. This result indicates that micro-aggregates can harbor more population of the pathogen.

PICRUST2 was used to predict the potential microbial functions of the microbial communities of different size classes in the fruiting stage. Predicted KEGG ORTHOLOGYs (KOs) were compared to determine the potential functions enriched in macro-versus micro-aggregates. As shown in Fig. 5d, macro-aggregates were relatively enriched with 22 KOs compared to micro-aggregates. For micro-aggregates, only five KOs were enriched. Interestingly, four of the KOs enriched in macro-aggregates are involved in the biosynthesis of antibiotics (i.e., K15320, K16421, K13547, and K16423 are involved in the biosynthesis of enediyne antibiotic, vancomycin, neomycin/kanamycin/gentamicin, and vancomycin, respectively). Moreover, two of the KOs enriched in macro-aggregates are related to antimicrobial resistance (i.e., K00561 and K18220). In contrast, metabolism KOs enriched in micro-aggregates are associated with phosphatase, amino sugar and nucleotide sugar, dibasic acid, and porphyrin, with no KOs associated with antibiotic biosynthesis or resistance.

## Discussion

There is a long history of research investigating root-adhering soil aggregation and its relationship with soil microbiota. For instance, it has been shown that wetting and drying cycles can impact rhizosphere soil aggregation [21], and rhizosphere microbiota, such as *Bacillus polymyxa* [44] and *Rhizobuim* sp. [45], can improve rhizosphere soil aggregation. In our experiment, we also found that soil aggregation was negatively affected by the growth of plant roots (Fig. 1b), which is in line with several arguments that plant roots can lead to the disruption of soil aggregates. Firstly, the tomato roots become longer as the growth of tomatoes and penetrate into the macropores, such a process may physically break the existing macro-aggregates [46]; Secondly, some of the organic compounds released by plant roots can increase the dispersion of clay particles [47]. In this experiment, the soil type was a clay loam, with a high clay content of 30% [48], in which the dispersion of clay can cause a breakdown of the macro-aggregates; Moreover, Wet-dry cycles caused by water uptake of tomato roots can disrupt soil aggregation in swelling clays, making the clay particles separate from other particles, decreasing the soil aggregate stability [49]. The development of tomato growth stages will increase the effect of water uptake throughout the growth of tomato roots, boosting the negative impact on soil aggregation.

Numerous studies have shown that the microbiomes of root-adhering soils are impacted by plant roots [50–54]. However, nearly all such research has relied on typical soil sampling strategies, i.e., taking the entirety of the root-adhering soil as a collective rhizosphere sample. Unfortunately, such sampling approaches do not account for the complex and heterogeneous nature of the soil matrix at the micro-scale, where different sizes of soil aggregates can provide an array of different micro-environments that may differ in their physical-chemical conditions. For example, larger soil aggregates were shown to have higher organic matter content and carbon stocks in Loess Plateau soils [55], which could support greater bacterial biomass within [56]; meanwhile, heavy metals such as Cd, Cr, Cu, Zn were found to have higher concentrations in smaller aggregates of farmland soil [57], these heavy metals are toxic to the vast majority of bacteria and impact their activity in organic matter degradation [58]. Macro-aggregates from natural grassland in China also had a lower pH and higher porosity than micro-aggregates [59], while soil pH is a critical driver of soil bacterial community structure [60, 61]. The typical soil sample is huge compared to individual soil aggregates. This discrepancy in scale at the level of the spatial habitats of individual microorganisms does not allow for the study of *in-situ* species interactions in microbial niches [18]. Similarly, although many studies have examined the influence of plant roots on the rhizosphere microbiome, little is known about how this influence is exerted across different root-adhering soil aggregate size classes. Our research shows the *in-situ* impact of fine-scale heterogeneity of soil aggregates on bacteriome assembly in the rhizosphere and how these changes throughout plant development. We found that succession within the bacterial communities across soil aggregate sizes differed, with micro-aggregates being more responsive to the changing impact of plant roots both at the community (i.e., total density, community diversity) to species levels (i.e., potential functional ASVs, pathogen *R. solanacearum*). Our results highlight the importance of fine-scale heterogeneity among soil aggregates on plant-soil feedback research, giving heed to the appeal that soil aggregation should be considered as an important driver of evolution and interaction in soil microbial communities [17, 62].

Plant roots can secrete a wide range and a large quantity (5–21% of total fixed carbon) of compounds into the rhizosphere, which can be assimilated by root-associated bacteria [63]. In this study, the planting of tomatoes also provides organic matter to soil-borne bacteria, causing an increase in bacterial density from the pre-planting to the seedling stage for all soil aggregate size classes, and this effect is most pronounced for micro-aggregates (Fig. 2a). This phenomenon may be attributed to the varying water storage capabilities of different soil aggregate sizes. Plant roots release organic compounds for microbes by root exudates [64]. The extent to which these liquids are acquired by soil aggregates may differ among size classes due to differences in pore sizes and structure within the soil: smaller size classes of soil aggregates exhibit reduced porosity [65] but possess a greater number of pores [66]. This, in turn, enhances the water retention capacity within micro-aggregates [67]. Consequently, micro-aggregates are better equipped to absorb and retain root exudates, thereby creating more favorable conditions for bacteria to utilize the nutrients derived from these exudates. This contributes to a higher overall bacterial density within micro-aggregates during the seedling stage. Conversely, the smaller porosity and stronger water storage capacity of micro-aggregates result in slower water flow within them. As a consequence, the influx of exudates into this category of aggregates is reduced. This, coupled with the depletion of initially safeguarded soil organic carbon, leads to a decline in total bacterial density within micro-aggregates during the flowering stage. Intriguingly, bacterial density experiences a renewed increase during the fruiting stage (Fig. 2a). This pattern aligns with the existing framework regarding the root-driven aggregates-turnover associated rhizosphere priming effect: plant roots can drive the release of aggregate-protected C for microbial decomposition and then facilitate new C (from root exudates) sequestration [68]. As a result, the total bacterial densities within micro-aggregates increased in the early growth stage of tomato and then decreased in the later stage due to the dynamic of soil organic matters.

In contrast, macro-aggregates (i.e., LMa and SMa) showed an opposite pattern in bacterial density with the highest bacterial densities at the flowering stage (Fig. 2b). This could be linked with the higher saturated hydraulic conductivity [65] and less protection of soil organic matter [69] by the larger size of soil aggregates, causing a slower dynamic of soil organic matter impacted by the rhizosphere priming effect. Bacterial densities thus show aggregate size class-dependent dynamics throughout the different growth stages of the plant [70]. Here, we used qPCR to track the changes in bacterial abundance, although the copy number of the 16 S rRNA gene may also reflect certain growth traits such as growth rate [71], the results from qPCR are still comparable, as it is a systematic error which similar to all the samples that be tested. Due to the limitations of technology, qPCR is still a commonly used approach for bacterial abundance determination, methodologies should be developed to improve the accuracy.

Previous studies have shown differences across root-adhering soil aggregates concerning soil respiration [72, 73], microbial biomass, and enzymatic activities [22]. Here, we provide additional information regarding bacterial community traits such as density, community structure, taxon distribution, and potential functioning as related to disease suppression. For instance, in the seedling stage, larger temporal changes were found in the smaller size of soil aggregates in community turnover (Fig. 3b) and more taxa significantly changed in relative abundance were found for these samples (Fig. 4). It may be that the root exudates and plant-derived substrates are binding agents of macro-aggregates [46]. Consequently, the chemical composition of nutrient resources derived from root-generated substrates could exhibit similarities to the organic matter present in macro-aggregates. As a consequence, bacteria relying on “old” organic material might have been subjected to more pronounced disturbances triggered by the introduction of root-derived substrates during the tomato’s seedling stage [51].

At the taxonomic level, the three sizes of soil aggregates only shared a few common increased ASVs, mostly classified as Proteobacteria (Fig. 4) in the seedling stage. This is in line with a previous report that showed that exogenous seedling tomato root exudates could increase the relative abundance of Proteobacteria [74]. Interestingly, the most significantly changed ASVs at the seedling stage are unique to individual soil aggregates (Fig. 4), indicating the importance of appreciating the fine-scale heterogeneity of soil aggregates.

Interestingly, we found a potential linkage between different bacteria within soil aggregates and potential pathogen inhibition function. Exposure to plant disease-causing agents can induce the recruitment of an assemblage of plant-beneficial bacteria in the rhizosphere, resulting in an enrichment of disease-suppressive microbial traits in the rhizosphere of disease-exposed plants [75]. Moreover, evidence has also shown that, as root-adhering soil is dislodged, such impacts on the soil microbiome can extend beyond the immediate plant surface after pathogen invasion, thereby inducing a soil-borne legacy to benefit subsequent plant generations [76]. The fields in our experiment suffered acutely from tomato *R. solanacearum* disease [29, 30], allowing examine the distribution of both the pathogen and potentially pathogen-suppressive bacteria across different size classes of root-adhering soil aggregates. The tomato bacterial wilt outbreak occurred at the fruiting stage in our experimental field. This corresponded to a large increase in the relative abundance of an ASV classified as the pathogen genus, *Ralstonia*, in the rhizosphere. We found an interesting distribution of the pathogen and potentially pathogen inhibition taxa across soil aggregates, where the pathogen is most prevalent in micro-aggregates (Fig. 5c), while more candidate pathogen inhibitors reside in macro-aggregates (Fig. 5b). These taxa, which are negatively correlated to the density of the *Ralstonia* ASV in the rhizosphere, were classified into groups that have previously been reported to be associated with disease suppression. For instance, some species of *Nocardioides* can produce antibiotic compounds that exhibit antibacterial activities toward Gram-positive and Gram-negative bacteria, such as the rice disease pathogen *Xanthomonas oryzae* [77]. The genus *Hyphomicrobium* was also reported to increase with Pine wilt disease resistance-inducing chemical elicitor acibenzolar-s-methyl in the rhizosphere [78]. Unfortunately, the other two ASVs, ASV122 and ASV556, could only be classified at the family level, making it more difficult to relate them to previous knowledge related to pathogen suppression. It should be noted that our results only suggest the potential function of these ASVs in disease suppression. Confirmation of such activities would require future isolation and inhibitions studies.

To get more insight into differentially represented microbial functions related to disease suppression across aggregate size classes, we performed PICRUST2 analysis to predict the potential functions of differentially distributed ASVs across soil aggregate size classes at the fruiting stage. Interestingly, the results of predicted metagenome properties (Fig. 5d) supported the results related to the taxa that were negatively related to the pathogen in macro-aggregates (Fig. 5b): K15320, K16421, K13547, K15956, and K16423 were enriched in macro-aggregates, with these KOs being involved in antibiotic biosynthesis including enediyne, vancomycin, Neomycin/kanamycin/gentamicin, daunorubicin, and vancomycin, respectively [70, 79–81]. Antibiotic resistance genes were also found to be enriched in macro-aggregates, K18353, K00561, and K18220 being associated with antimicrobial resistance [82–84]. These results suggest that macro-aggregates represent habitats with relatively high levels of antibiotics, which is in line with the finding that pathogen densities are highest in micro-aggregates. To develop a more complete picture of differential pathogen inhibition capabilities across soil aggregate size classes, additional studies will be needed to track the densities of genes, transcripts, and/or proteins involved with antibiotic production and resistance. The higher abundance of *R. solanacearum* in micro-aggregates indicates its preference for micro-environments in such size classes (Fig. 5c). This preference might be linked to the stronger water storage capacity of micro-aggregates [67], while higher soil moisture can support a greater density of *R. solanacearum* [85]. More populations of potential pathogen-inhibiting taxa (Fig. 4a,b) and predicted antibiotics genes (Fig. 5d) were found in macro-aggregates. This might be induced by the amendment of organic fertilizers, which was shown to increase disease suppressiveness by triggering beneficial microbes [86], and such “fresh” organic matters would first format into macro-aggregates according to the existing soil aggregate turnover model [46], resulting in a direct simulating on inhibitors within this size classes.

Our findings suggest that bacterial community succession and functioning are dependent on soil aggregate size class, with micro-aggregates being more favorable to the soil-borne pathogen *R. solanacearum* than macro-aggregates. Moreover, we found that soil aggregation is influenced quickly and substantially by agricultural management, creating more pathogen-favored habitats (i.e., micro-aggregates) in the tomato field. This might be the major factor causing the tomato consecutive cropping obstacle. Potential management strategies could be developed for a more sustainable tomato continuous cropping agricultural system according to our study: (1) The improvement of the macro-aggregate proportion and the increase of macro-aggregate stability can help decrease the amount of habitat for the pathogen and increase the abundance of pathogen inhibitors; (2) the inoculation of micro-aggregates favored pathogen-inhibit microorganisms may contribute to the decrease of pathogen density in soils. As our study is limited to a single experimental field, with its specific soil properties, future research should be dedicated to examining similar aggregate-specific properties across other soil types and management schemes. This knowledge related to the properties and activities of soil aggregate samples could help us to better understand the microbial processes and interactions that determine disease suppression and outbreak and to develop appropriate management strategies.

## Conclusion

In summary, our study suggests that the soil structure is negatively impacted by the plant root throughout tomato growth stages and highlights the importance of fine-scale heterogeneity of soil aggregate size classes in modulating the effect of plant roots on bacterial community characteristics and soil-borne disease-associated functions. Distinct patterns were observed across different soil aggregate size classes, revealing variations in total density dynamics, shifts in community composition, and alterations in the abundance of taxa. Remarkably, the tomato bacterial wilt pathogen exhibited a preference for micro-aggregates, while taxa negatively correlated with pathogen abundance in the rhizosphere, and predicted functional genes associated with antibiotics were found to be more prevalent within macro-aggregates. These insights accentuate the intricate interplay between plant root effects, bacterial community traits, and functions relevant to disease management within the context of soil structure, which suggests that better management of soil structure such as improvements of soil aggregation and macro-aggregates stability, pathogen-suppressing microorganisms preferring micro-aggregates can significantly contribute to the sustainability of continuous cropping agricultural system.

### Supplementary information

Supplementary Information

## Data Availability

16 S rRNA gene amplicon sequence data is available in the National Center for Biotechnology Information (NCBI: https://www.ncbi.nlm.nih.gov/) with the BioProject accession number PRJNA911225.
