# Supplementary material for: Tomato growth stage modulates bacterial communities across different soil aggregate sizes and disease levels
Source: ISME Commun. 2023 Sep 26;3:104. doi: 10.1038/s43705-023-00312-x (PMC10522649; doi:10.1038/s43705-023-00312-x)
Supplement: Supplementary file 1 — Supplementary Information [file 43705_2023_312_MOESM1_ESM.docx]

**Supplementary Information**

PCR amplification for sequencing

PCR amplifications were performed using Q5® High-Fidelity DNA Polymerase (M0491L, New England Biolabs Inc., The Netherlands) with the following mixture: 5×reaction buffer 5 μL, 5×GC buffer 5μL, dNTP (2.5 mM) 2 μL, forward primer (10 μM) 1 μL, reverse primer (10 μM) 1μL, DNA template 2μL, ddH2O 8.75 μL. The following thermocycling program was used: initial denaturation 98 ℃ 2 min, denaturation 98 ℃ 15 s, annealing 55 ℃ 30 s, extension 72 ℃ 30 s, final extension 72 ℃ 5 min, 10 ℃ hold. 25-30 cycles. Reactions were run in a 2720 thermal cycler (Applied Biosystems, Life Technologies, CA, US)

Rhizosphere amplicon sequencing data

We used rhizosphere amplicon sequencing data from Zhao (2020) to identify the ASV that corresponds to the pathogen of tomato Ralstonia disease and its potential inhibitors in the rhizosphere, which allowed us to investigate their distribution across soil aggregate size classes. The rhizosphere samples were the soils that remained on the tomato roots after being shaken, they were collected by root wash and centrifugation. Rhizosphere samples were performed with DNA extraction and amplicon sequencing in the same protocol as root-adhering soil aggregate samples.

qPCR protocol

For bacterial density, part of the V3 region of the 16S rRNA gene (primer 338F 5’-ACTCCTACGGGAGGCAGCAG-3’ and primer 518R 5’-ATTACCGCGGCTGCTGG-3’) were amplified with a reaction mixture of 10 μL SYBR Premix Ex Taq™ (2×), 0.4 μL of each primer (10 pmol/μL), 0.4 μL ROX Reference Dye II, and 1 μL of template DNA (20 ng/μL) with a final volume of 20 μL.

For *R. solanacearum* density, *filC* gene (primer F 5’-GAACGCCAACGGTGCGAACT-3’ and primer R 5’-GGCGGCCTTCAGGGAGGTC-3’) was amplified with a reaction mixture of 10 μL SYBR Premix Ex Taq™ (2×), 0.8 μL of each primer (10 pmol/μL), 0.4 μL ROX Reference Dye II, and 1 μL of template DNA (20 ng/μL) with a final volume of 20 μL. SYBR Premix Ex Taq^TM^ (Cat# RR420A, TaKaRa Biotechnology Co., Ltd). All qPCR analyses were performed on each of the 6 biological replicates, and each involved three technical replicates with three negative controls (ddH_2_O as template).

All qPCR was performed on an Applied Biosystem Step One Plus Real-Time PCR System (Applied Biosystems, Life Technologies, CA, US) using a program of initially denaturing for 30 s at 95 °C, followed by cycling for 40 times with a 5s denaturalizing step at 95 °C and a 34s extension at 60 °C. Melt curve analysis followed a standard protocol used to identify the characteristic peak of the PCR product.

Fig S1


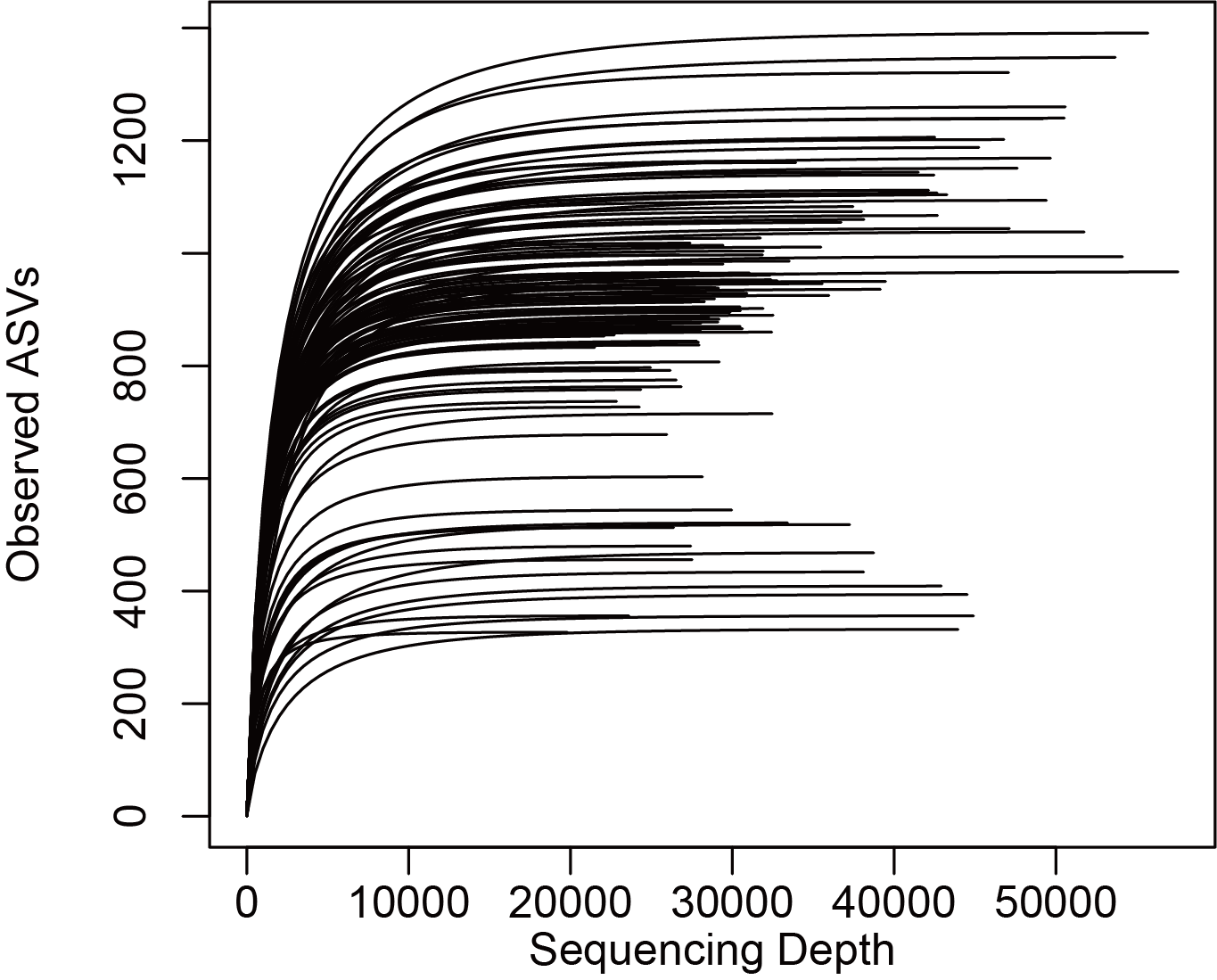


**Fig S1. Rarefaction curve of observed ASVs in all samples.**

Fig S2


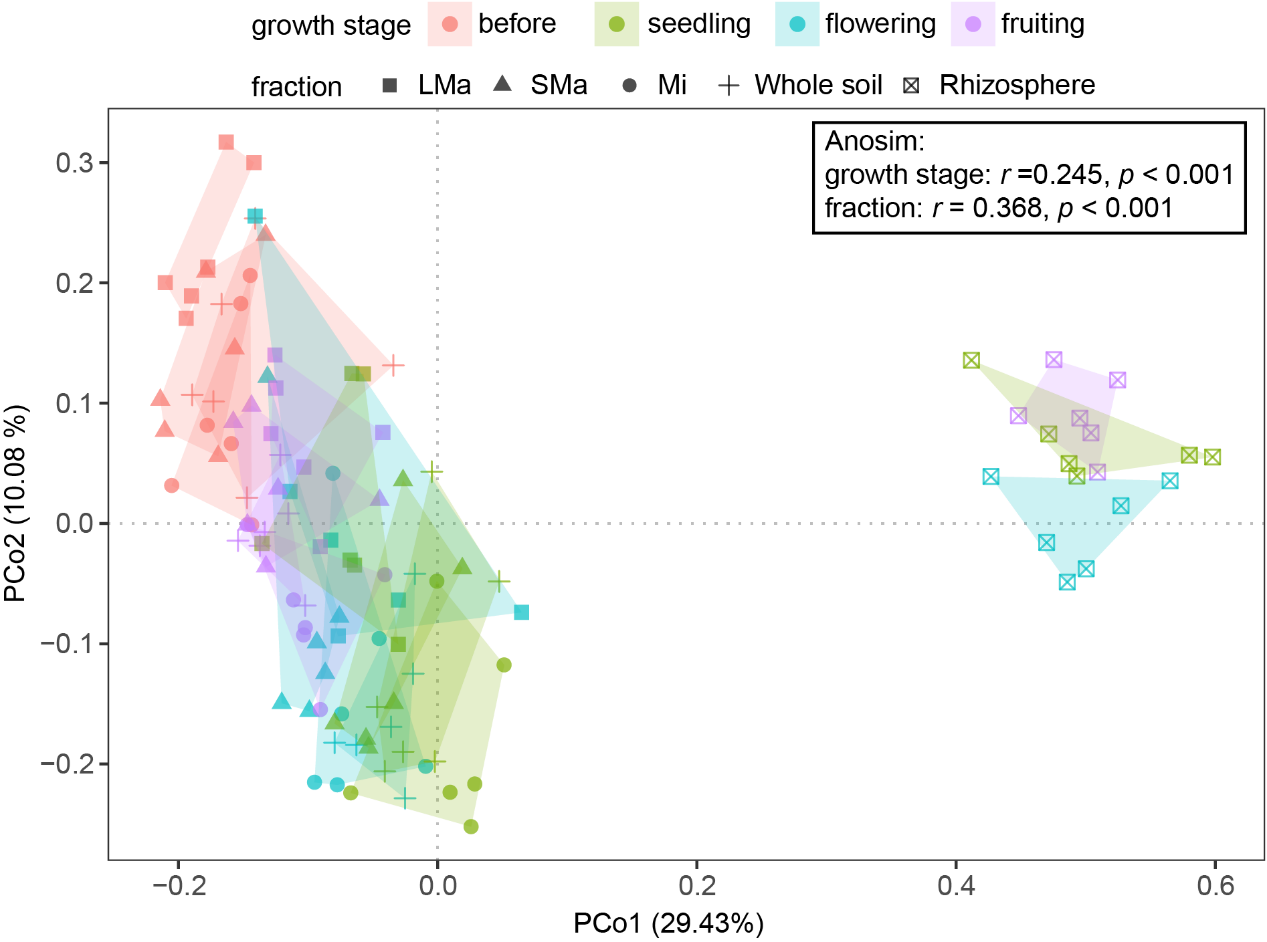


**Fig S2 Principal coordinate analysis (PCoA) of bacterial composition in all samples based on Bray-Curtis distance.** LMa, large macro-aggregate, 1-2 mm; SMa, small macro-aggregate, 0.25-1 mm; Mi, micro-aggregate, < 0.25 mm.

Fig S3


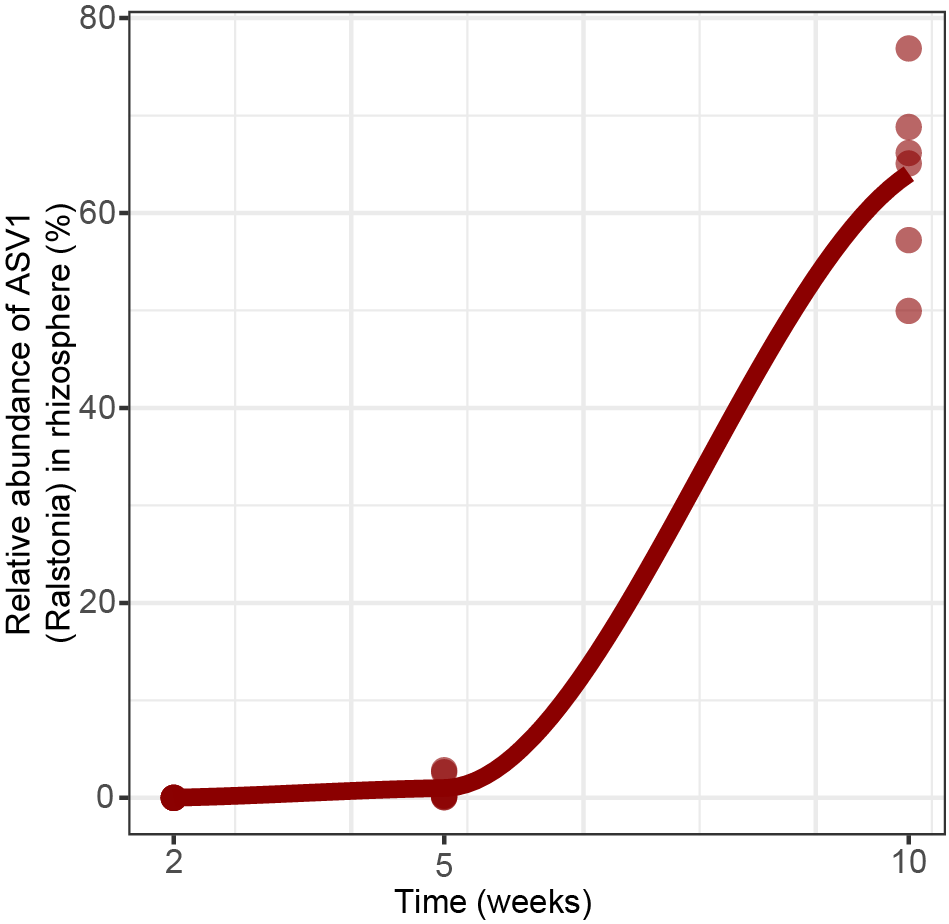


**Fig S3 Relative abundance of ASV1 that classified as *Ralstonia* genus in rhizosphere throughout tomato growth stages.**

Fig S4


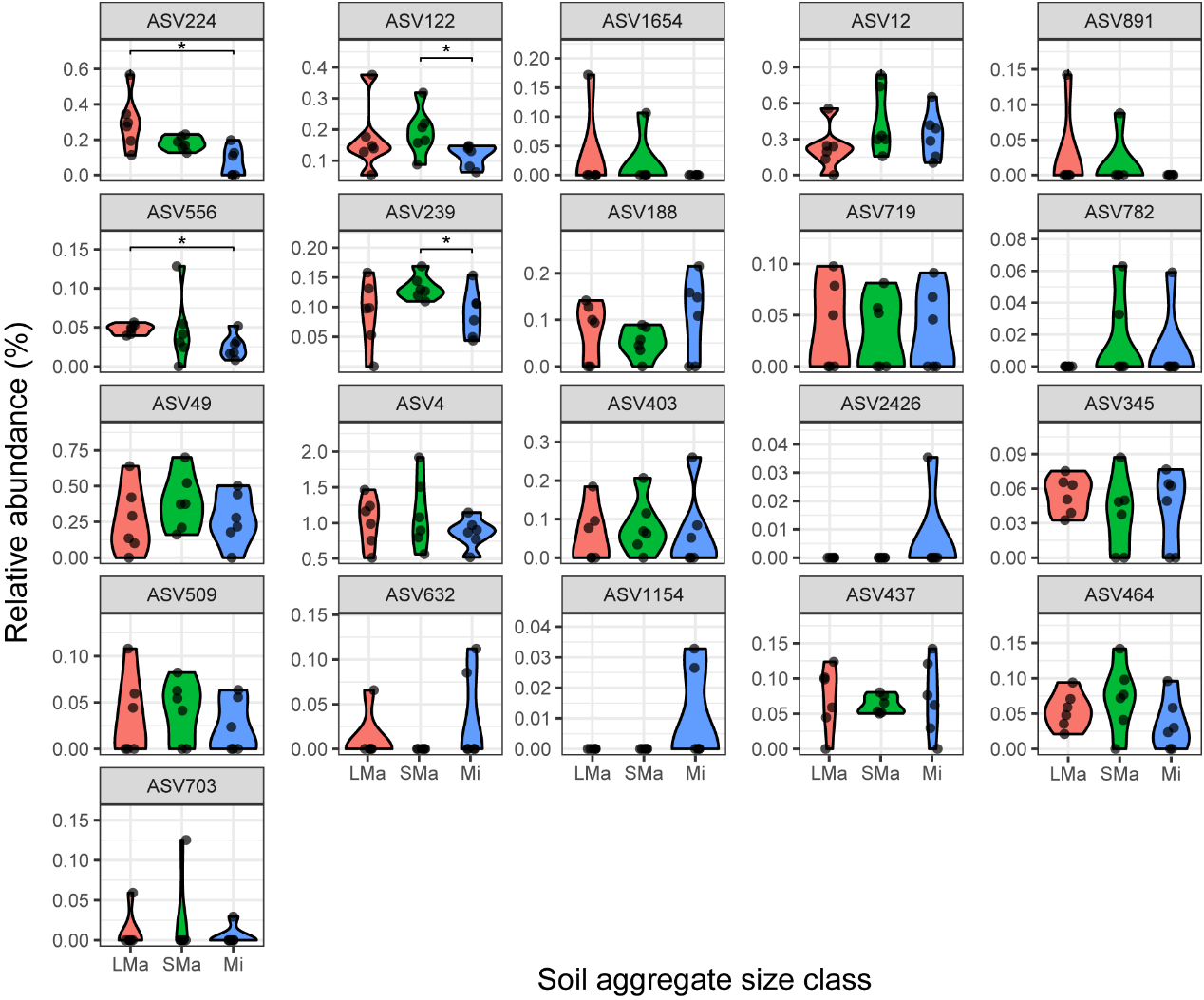


**Fig S4 Distribution of pathogen negatively correlated ASVs among soil aggregate size classes.** LMa, large macro-aggregate, 1-2 mm; SMa, small macro-aggregate, 0.25-1 mm; Mi, micro-aggregate, < 0.25 mm. Significance was determined by Wilcoxon Rank Sum and Signed Rank Tests: ns, no significant difference; *, *p* < 0.05.
